# SARS-CoV-2 spike protein causes cardiovascular disease independent of viral infection

**DOI:** 10.1042/CS20220028

**Published:** 2022-03-29

**Authors:** John D. Imig

**Affiliations:** Drug Discovery Center, Cardiovascular Center, Medical College of Wisconsin, Milwaukee, WI, U.S.A.

**Keywords:** cardiovascular disease, CD147 Receptor, COVID-19, Pericytes, virus spike protein

## Abstract

The SARS-CoV-2 virus that results in COVID-19 has been found to damage multiple organs beyond the lung. Interestingly, the SARS-CoV-2 spike (S) protein can be found circulating in the blood of COVID-19 patients. Experimental findings are demonstrating that the circulating S protein can bind to receptors resulting in inflammation and cell, tissue, and organ damage. Avolio et al. previously determined that the S protein acting through the cluster of differentiation 147 (CD147) receptor, and another unknown mechanism had detrimental effects on human cardiac pericytes (*Clin Sci (Lond)* (2021) 135 (24): 2667–2689. DOI: 10.1042/CS20210735). These findings support the notion that circulating SARS-CoV-2 S protein could contribute to cardiovascular disease independent of viral infection. Future studies are needed to determine the effect of the S protein on pericytes in other organs and evaluate the effectiveness of CD147 receptor-blocking therapies to decrease organ damage caused by the S protein.

COVID-19 has been determined to increase mortality in patients with underlying cardiovascular conditions [1,2]. The increase in cardiovascular events in COVID-19 patients has been widely thought to be mediated by infection by SARS-CoV-2 and uptake by angiotensin-converting enzyme-2 (ACE2) [1,2]. Intriguingly, Avolio et al. in this edition of *Clinical Science* found that circulating SARS-CoV-2 spike (S) protein in the circulation can cause detrimental effects on cardiac pericytes independent of viral infection [3]. Cardiac pericytes are mural cells that support the maintenance and repair of the vasculature in the heart [4]. The study by Avolio et al. determined that the S protein actions on the cardiac pericytes are through cluster of differentiation 147 (CD147)-receptor mediated signaling [3]. Thus, blocking the CD147 receptor in COVID-19 patients could decrease cardiovascular disease caused by the circulating S protein.

We have reached the 2-year anniversary for the COVID-19 pandemic, and it is clear that SARS-CoV-2 will become an endemic virus. As such, the long-term consequences of SARS-CoV-2 on multiple organ systems needs to be evaluated. Cardiovascular disorders such as myocardial infarction, arrhythmias, and thromboembolism appear to be a consequence of COVID-19 [1]. The mechanisms by which COVID-19 causes or enhances cardiovascular diseases have been under intense investigation [1,2,5]. A potential mechanism for cardiac disease in COVID-19 has been myocardial involvement mediated by ACE2 [1,2,5]. The cytokine storm and hypoxia-induced actions leading to cardiomyocyte apoptosis is another mechanism suggested for cardiovascular mortality in COVID-19 patients [5,6]. Experimental studies by Avolio et al. demonstrated the presence of the SARS-CoV-2 S protein in the peripheral blood of COVID-19 patients [3]. Findings in this study determined that close to 70% of the patients did not have their pericytes infected by SARS-CoV-2 [3]. It was further demonstrated that the S protein was capable of increasing cardiac pericyte migration, reduced endothelial cell network formation in Matrigel, induced pericyte cytokine secretion, and increased production of pro-apoptotic factors leading to endothelial cell death [3]. Taken together, COVID-19 studies demonstrate that SAR-CoV-2 infection via ACE2 and the circulating S protein independent of infection can lead to cardiovascular dysfunction.

The concept that the S protein can cause detrimental effects in COVID-19 patients independent of infection could partially explain the long-term health issues. A previous study demonstrated that the S protein could damage the endothelium and disrupt the blood–brain ba[rrier resulting in perivascular inflammation [7–9]. Other actions of the S protein include direct stimulation of peripheral nerves, release of inflammatory factors, and stimulation of vasoactive mediators such as platelet-activating factor (PAF) [2,7]. Human epithelial cells demonstrated increased senescence and inflammation following transfection with the S protein [10]. These findings clearly implicate the S protein as mediating cellular dysfunction; however, the receptors and signaling mechanisms vary depending on the cell type.

Several receptors have been implicated in SAR-CoV-2 infection and S protein actions in COVID-19 patients. Even though ACE2 appears to be the primary receptor that binds the S protein of SARS-CoV-2 to cause infection, there is evidence that CD147, neuropilin-1, dipeptidyl peptidase 4, alanyl aminopeptidase, and glutamyl aminopeptidase can act as receptors for the S protein [11,12]. The findings of Avolio et al. clearly determined that the S protein binds to CD147 in human cardiac pericytes [3]. CD147 is transmembrane protein also referred to as basigin of extracellular matrix metalloproteinase inducer (EMMPRIN) [11,12]. CD147 was previously found to be the epithelial cell receptor for the measles virus [11,12]. Moreover, the concentration of CD147 is elevated in patients with inflammation and diabetes and asthmatic complications [11]. Several signaling pathways are thought to contribute to CD147 actions. These signaling pathways include MAPK p38, ERK-1/2, and NF-κB [11,12]. Activation of CD147 mediates macrophage inflammation, induction of MMP-9 expression, and cytokine expression in endothelial cells [13]. Experimental studies by Avolio et al. used a CD147 blocking antibody or mRNA silencing approach to determine that the S protein activation of cardiac pericyte CD147 receptors resulted in ERK1/2 signaling to exert detrimental effects [3]. Interestingly, the effects of the S protein to secrete proinflammatory cytokines to induce endothelial cell death occurred by a mechanism independent of the CD147 receptor [3]. The identity of the proinflammatory actions of the S protein on pericytes remains to be determined.

The finding that CD147 receptor can mediate non-infective COVID-19 microvascular disease has therapeutic implications. A humanized anti-CD147 antibody that has been examined in SARS-CoV-2 pneumonia is meplazumab [14]. Meplazumab inhibits the interaction between CD147 and the S protein in a dose-dependent manner [14]. The condition and recovery rate of patients with COVID-19 pneumonia was improved with meplazumab treatment while maintaining a safety profile [14]. Other humanized anti-CD147 antibodies such as metuximab and metuzamb have been developed and found to have a favorable safety profile; however, their effects in COVID-19 are less well characterized [11,14]. Another therapeutic candidate for decreasing the actions of the S protein on the CD147 receptor is statins [11,15]. Statins regulate CD147 levels and induce changes in the CD147 structure, function, and expression via blocking N-glycosylation and isoprenylation of the proteins [15]. Investigations have demonstrated that statins decrease the levels of CD147 in human cells and can be used as a co-adjuvant treatment for COVID-19 management [16]. Lastly, azithromycin a macrolide antibiotic which inhibits bacterial protein synthesis is thought to reduce viral loads in COVID-19 patients by interfering with S protein CD147 interactions [14]. Future investigations and clinical studies are needed to determine the effectiveness of CD147 treatments to combat cardiovascular disease in COVID-19 patients.

## Conclusion

**Figure 1:**
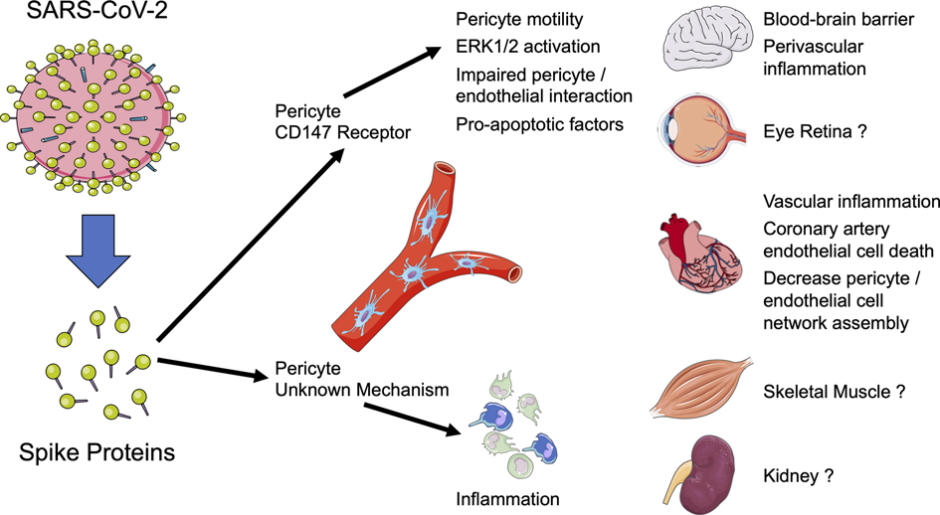
SARS-CoV-2 S protein’s actions on pericytes *Left:* SARS-CoV-2 can shed S proteins into the blood. *Middle:* S protein in the blood can activate CD147 receptors on pericytes to increase pericyte motility, activate pericyte ERK 1/2, cause impaired pericyte/endothelial cell interactions, and cause pericytes to release pro-apoptotic factors. Pericytes also release pro-inflammatory factors via an unknown mechanism. *Right:* S protein actions on pericytes causes damage to the brain and cardiovascular system while detrimental effects on the eye, skeletal muscle, and kidney remain unknown.

As the COVID-19 marches on from pandemic status to endemic status, the impact of the SARS-CoV-2 virus on cardiovascular health needs to be better understood. There has been extensive evaluation of SARS-CoV-2 infection and COVID-19 on cardiovascular health [1,2]; however, there is emerging evidence that the S protein shed from SARS-CoV-2 can circulate in the blood of patients and have detrimental consequences [3,10,11]. (Figure 1) The study by Avolio et al. in this issue of *Clinical Science* provides strong evidence that the circulating S protein could be more detrimental to cardiac health than infection of SARS-CoV-2 to the heart [3]. More specifically, the S protein was found to act through the CD147 receptor on human cardiac pericytes to cause microvascular dysfunction [3]. The authors also determined that the S protein caused human cardiac pericyte inflammation via yet to be determined mechanisms [3].

Findings in the present study have implications for other organ systems that could be affected by the S protein and therapeutics to combat the detrimental actions of the S protein. The fact that pericytes are found in other organs such as brain, eye, skeletal muscle, and kidney suggest that the circulating S protein could cause damage to these organs [11,17,18]. The impact of the S protein on the brain has determined that it can disrupt the blood–brain barrier and cause perivascular inflammation [7–9]. Pathological consequences of the circulating S protein on pericytes in other organ systems remain to be investigated. In addition, therapeutics used to block or decrease expression of the CD147 receptor could be effective against cardiovascular disease and other organ diseases associated with SARS-CoV-2 virus and COVID-19. Therapies that are being evaluated that impact the CD147 receptor include humanized anti-CD147 antibodies, statins, and azithromycin [11,14,15].

As variants of the S protein emerge such as Omicron it will be important to continue to evaluate the potential pathological consequences of circulating S protein. The current study by Avolio et al. found similar results with the Alpha and Delta variants of the S protein on human cardiac pericytes [3]. Although the findings of the current study are provocative, future investigations need to expand the doses of S proteins to lower levels and other S protein variants for *in vitro* studies. Lastly, as we investigate SARS-CoV-2 and the circulating S protein and the impact on human health, we will be better prepared to treat patients as COVID-19 moves from a pandemic to an endemic status.

## Abbreviations

ACE2: angiotensin-converting enzyme-2
CD147: cluster of differentiation 147
S: spike protein
